# ALY proteins participate in multifaceted Nep1_Mo_-triggered responses in *Nicotiana benthamiana* and *Arabidopsis thaliana*

**DOI:** 10.1093/jxb/eru136

**Published:** 2014-04-10

**Authors:** Wenjun Teng, Huajian Zhang, Wei Wang, Deqing Li, Meifang Wang, Jiewen Liu, Haifeng Zhang, Xiaobo Zheng, Zhengguang Zhang

**Affiliations:** ^1^Department of Plant Pathology, College of Plant Protection, Nanjing Agricultural University, Key Laboratory of Monitoring and Management of Crop Diseases and Pest Insects, Ministry of Agriculture, Nanjing, 210095, China; ^2^Department of Plant Pathology, College of Plant Protection, Anhui Agricultural University, Hefei, 230036, China

**Keywords:** ALY, disease resistance, hypersensitive response, Nep1, nitric oxide, stomatal closure.

## Abstract

Previously, it was found that Nep1_Mo_ (a Nep1-like protein from *Magnaporthe oryzae*) could trigger a variety of plant responses, including stomatal closure, hypersensitive cell death (HCD), and defence-related gene expression, in *Nicotiana benthamiana*. In this study, it was found that Nep1_Mo_-induced cell death could be inhibited by the virus-induced gene silencing of *NbALY916* in *N. benthamiana*. *NbALY916*-silenced plants showed impaired Nep1_Mo_-induced stomatal closure, decreased Nep1_Mo_-induced production of hydrogen peroxide (H_2_O_2_) and nitric oxide (NO) in guard cells, and reduced Nep1_Mo_-induced resistance against *Phytophthora nicotianae*. It also found that the deletion of *AtALY4*, an orthologue of *NbALY916* in *Arabidopsis thaliana*, impaired Nep1_Mo_-triggered stomatal closure, HCD, and defence-related gene expression. The compromised stomatal closure observed in the *NbALY916*-silenced plants and At*ALY4* mutants was inhibited by the application of H_2_O_2_ and sodium nitroprusside (an NO donor), and both Nep1_Mo_ and H_2_O_2_ stimulated guard cell NO synthesis. Conversely, NO-induced stomatal closure was found not to require H_2_O_2_ synthesis; and NO treatment did not induce H_2_O_2_ production in guard cells. Taken together, these results demonstrate that the NbAlY916/AtAlY4–H_2_O_2_–NO pathway mediates multiple Nep1_Mo_-triggered responses, including stomatal closure, HCD, and defence-related gene expression.

## Summary

The NbAlY916/AtAlY4–H_2_O_2_–NO pathway mediates multiple Nep1_Mo_-triggered responses, including stomatal closure, hypersensitive cell death, and defence-related gene expression.

## Introduction

Plants have evolved multiple defence mechanisms against phytopathogens, including the hypersensitive response (HR) and stomatal closure (Dangl *et al.*, 1996; Lam *et al.*, 2001; Mur *et al.*, 2008). The HR is considered to be a form of localized hypersensitive cell death (HCD) that results in the formation of necrotic lesions around sites of infection (Hammond-Kosack and Jones, 1996; Greenberg and Yao, 2004). HCD is initiated upon plant–pathogen recognition. In addition to classic gene–gene interactions, the binding of selected pathogen-associated molecular patterns (PAMPs) and elicitors to the receptors can also trigger the HR, leading to PAMP-triggered immunity (PTI) (Nurnberger *et al.*, 2004; Schwessinger and Zipfel, 2008).

**N**ecrosis and the secretion of **e**thylene-inducing **p**eptide 1 (Nep1) occurs in taxonomically diverse organisms, including bacteria, fungi, and oomycetes (Gijzen and Nürnberger, 2006). In some cases, microbial Nep1-like proteins (NLPs) are considered to be positive virulence factors that accelerate disease and pathogen growth in host plants through disintegration (Amsellem *et al.*, 2002; Pemberton *et al.*, 2005; Ottmann *et al.*, 2009). However, NLPs trigger cell death and immune responses in many dicotyledonous plants (Pemberton and Salmond, 2004; Gijzen and Nurnberger, 2006; Schwessinger and Zipfel, 2008). It is believed that NLPs may associate with the outer surface of the plasma membrane to trigger the HR (Qutob *et al.*, 2006; Schouten *et al.*, 2008), and global gene expression analyses have revealed overlap between the responses to Nep1 and responses to other elicitors that lead to active ethylene and oxygen production (Bae *et al.*, 2006). It was recently demonstrated that the mitogen-activated protein kinase (MAPK) cascade is involved in Nep1_Mo_ (Nep1 from *Magnaporthe oryzae*)-triggered plant responses, and the MAPK signalling associated with HCD exhibits shared and distinct components with that of stomatal closure (Zhang *et al.*, 2012*a*). Bioinformatics and functional genetic screens have been used to identify and characterize genes mediated by such elicitor signalling. However, information on the signalling network is still fragmentary, and many important players involved in Nep1-induced plant immunity remain to be discovered.

High-throughput down-regulated expression screening of a plant cDNA library was previously performed using virus-induced gene silencing (VIGS), and several genes that suppress cell death in *Nicotiana benthamiana* leaves upon elicitor treatment were identified. The involvement of respiratory burst oxidase homologue (RBOH), vacuolar processing enzyme (VPE), G proteins, and MAPKs in elicitor-triggered plant immunity was demonstrated by *Potato virus X* (PVX)-based VIGS (Zhang *et al*., 2010, 2012*b*). Here, a cDNA identified in the screen, which encodes an Ally of AML-1 and LEF-1 (ALY) protein that acts as a suppressor of Nep1_Mo_-mediated immunity in *N. benthamiana* and *Arabidopsis thaliana*, is reported. The ALY family is a group of plant RNA-binding proteins. Animals encode only one or two ALY proteins, whereas *A. thaliana* encodes four ALYs; in comparison, many monocots encode four or more ALYs (Uhrig *et al.*, 2004; Canto *et al.*, 2006). Animal (mouse, *Drosophila*, and human) and yeast ALY proteins contribute to the export of mRNAs from the nucleus before translation, and join with the exon junction complex of proteins marking mRNAs in the course of splicing (Bruhn *et al.*, 1997; Zhou *et al.*, 2000; Storozhenko *et al.*, 2001). Animal ALY proteins are also known as transcriptional co-activators, and promote the interaction of DNA-binding proteins. However, the function of plant ALYs is unknown.

Here, by employing VIGS, it was found that *NbALY916* is involved in the regulation of Nep1_Mo_-induced stomatal closure, HCD, and pathogen resistance in *N. benthamiana*. It is also demonstrated that AtALY4, an orthologue of NbALY916 in *A. thaliana*, plays the same role in Nep1_Mo_-triggered stomatal closure, HCD, and defence-related gene expression.

## Materials and methods

### Plant materials, elicitors, and treatment protocol

*Nicotiana benthamiana* and *Arabidopsis* were grown in a growth chamber under a 16/8h light/dark cycle at 25 °C. The T-DNA insertion line for *AtALY4* used in this study was At*ALY4* (CS331800) supplied by the Arabidopsis Resource Center (http://www.arabidopsis.org). The PCR primers (P1, GGGCATCAGGAGTTGAAGTT; P2, GGATCCCATAGATCCCATGA; and LBa1, GCGTGGAC CGCTTGCTGCAACT) were used to check the T-DNA insertions. To prepare Nep1_Mo_, overnight cultures of *Escherichia coli* BL21 cells carrying pET32b harbouring the *Nep1*_*Mo*_ gene (GenBank accession no. MGG_08454) were diluted (1:100) in Luria–Bertani medium containing ampicillin (50mg ml^–1^) and incubated at 37 °C for ~3h. When the OD_600_ of the culture reached 0.6, Nep1_Mo_ secretion into the culture medium was induced via the addition of 0.4mM isopropyl-β-d-thiogalactopyranoside for 6h. Nep1_Mo_ was expressed as a His-tag fusion protein. Protein purification was performed with Ni-nitrilotriacetic acid resin (QIAGEN, Valencia, CA, USA), and the purified proteins were dialysed against a phosphate-buffered saline (PBS) buffer (pH 7.4) and stored at 20 °C prior to use. Protein concentration was determined using the Bradford reagent (Qutob *et al.*, 2006), and concentrated stock solution (500nM) was prepared.

### DNA constructs and seedling infection for virus-induced gene silencing

To amplify a cDNA encompassing the entire open reading frame of NbALY916, the full-length cDNA for *NbALY916* was identified with RACE (rapid amplification of cDNA ends) using a SMART RACE amplification kit (BD Bioscience-Clontech, Palo Alto, CA, USA). VIGS for the *NbALY916* gene (GenBank accession no. AM167906) in *N. benthamiana* was performed using PVX, as previously described by Zhang *et al.* (2009, 2010). The *NbALY916* insert was 450bp, which was derived from the 3′ termini of its open reading frame and inserted into the PVX vector in the antisense direction to generate PVX.NbALY916. The construct containing the insert was transformed into *Agrobacterium tumefaciens* strain GV3101. Bacterial suspensions were applied to the undersides of *N. benthamiana* leaves using a 1ml needleless syringe. Plants exhibited mild mosaic symptoms 3 weeks after inoculation. The third or fourth leaf above the inoculated leaf, where silencing was most consistently established, was used for further analyses.

### Diaminobenzidine (DAB) staining

Following the methods of Thordal-Christensen *et al*. (1997), leaves were harvested 3h after Nep1_Mo_ treatment and immediately vacuum-infiltrated for 20min with PBS (pH 7.4) containing 0.5% (w/v) DAB. The leaves were placed in light for 10h and then boiled for 20min in 80% ethanol. Quantitative scoring of hydrogen peroxide (H_2_O_2_) staining in leaves was analysed using Quantity One software (BIO-RAD, Segrate, Italy).

### RNA isolation, semi-quantitative transcription–PCR (RT-PCR), and quantitative RT–PCR

The leaf fragments after Nep1_Mo_ inoculation were used for total RNA extraction using the Trizol reagent (TaKaRa, Dalian, China) following the manufacturer’s protocol and then treated with RNase-free DNase (TaKaRa). qRT–PCR was performed on the ABI 7300 Real-Time PCR System (Applied Biosystems, Foster City, CA, USA) and SYBR Premix Ex **Taq^™^**(TaKaRa, Dalian, China) according to the manufacturer’s instructions. The *N. benthamiana* genes (*NbEF1α* and *NbActin*) and *Arabidopsis* genes (*AtEF1α* and *AtS16*) were used as the internal reference genes to standardize the RNA sample for evaluating relative expression levels. For qRT–PCR assays, three independent biological samples were used, with each repetition having three technical replicates with a gene-specific primer (Supplementary Table S1 available at *JXB* online)

### Stomatal aperture measurements

Stomatal apertures were measured as described by Chen *et al.* (2004) and Zhang *et al.* (2009, 2010). Leaves were derived from PVX Nb, *NbALY916*-silenced plants, Col-0, and At*ALY4*. Abaxial (lower) epidermises were peeled off and floated in 5mM KCl, 50mM CaCl_2_, and 10mM MES-TRIS (pH 6.15) in light for 3h to open the stomata fully before experimentation to minimize the effects of other factors in stomatal response, because the mesophyll signals can also significantly influence stomatal behaviour. The epidermal strips then were followed by Nep1_Mo_ (50nM), sodium nitroprusside [SNP; a nitric oxide (NO) donor, 25 mM], and H_2_O_2_ (800 μM), respectively, to induce a stomatal response. For cPTIO [2-(4-carboxyphenyl)-4,4,5,5-tetramethylimidazoline-1-oxyl-3-oxide; an NO scavenger] treatment, cPTIO (400 μM) was applied for 1h prior to SNP treatment. The maximum diameter of stomata was measured under an optical microscope. At least 50 apertures in each treatment were obtained and the experiments were repeated three times.

### NO measurement in guard cells

NO accumulation was determined using the fluorophore 4,5-diaminofluorescein diacetate (DAF-2DA, Sigma-Aldrich) according to Ali *et al.* (2007). Epidermal strips were prepared from control and gene-silenced plants, and incubated in 5mM KCl, 50mM CaCl_2_, and 10mM MES-TRIS (pH 6.15) in the light for 2h, followed by incubation in 20 μM DAF-2DA for 1h in the dark at 30 °C, 65rpm, and finally rinsed three times with 10mM TRIS-HCl (pH 7.4) to wash off excess fluorophore. The dye-loaded tissues were treated with Nep1_Mo_ (50nM), SNP (25mM), and H_2_O_2_ (800 μM) for 1h, respectively. And then the fluorescence of guard cells was imaged (excitation wavelength, 470nm; emission wavelength, 515nm; Leica DMR, Germany) and analysed using Quantity One software.

### ROS measurement in guard cells

According to the method described by Pei *et al.* (2000), reactive oxygen species (ROS) measurement in guard cells was detected by using 2′,7′-dichlorofluorescein diacetate (H_2_DCFDA). Epidermal peels were ﬂoated in 5mM KCl, 50mM CaCl_2_, and 10mM MES-TRIS (pH 6.15) for 2h under light to induce stomatal opening, followed by incubation with 50 μM H_2_DCFDA for 10min and washing for 20min with incubation buffer. Images of guard cells were obtained 1h after Nep1_Mo_ treatment under a fluorescence microscope (excitation wavelength, 484nm; emission wavelength, 525nm; Leica DMR, Germany). Fluorescence emission from guard cells was analysed using Quantity One software.

### Disease resistance assay

One half (right side) of a leaf from PVX Nb and *NbALY916*-silenced *N. benthamiana* was infiltrated with either PBS (10nM) orNep1_Mo_ (50nM). Three hours later the leaves were collected and transferred to Petri dishes containing sterile water-saturated filter paper. A 9 mm×9mm hyphal plug of *Phytophthora nicotianae* was then placed on the surface of the left side of each leaf, which had not been infiltrated with Nep1_Mo_ or PBS. Samples were kept in the dark at 25 °C. Pictures of the lesions were taken at 48h post-inoculation, Leaves then were fixed with 100% ethanol. Resistance evaluation was based on the measurement of the diameter of *P. nicotianae* lesion size: inhibition=(diameter of control–diameter of elicitor)/diameter of control 100%. Data are means ±SE from three experiments.

Fully expanded Col-0 and At*ALY4* mutant leaves collected 3h after Nep1_Mo_ treatment (25 μl) were infiltrated with *Pseudomonas syringae* pv. *tomato* DC3000 (*Pst* DC3000) suspension (10^8^ cfu ml^–1^). Necrotic lesions caused by *Pst* DC3000 on leaves were observed. Leaves of inoculated plants were harvested and sterilized in 70% ethanol, and homogenized in sterile water. Bacteria were recovered on L-agar medium and induced resistance was deﬁned based on decreases in symptom severity and *in planta* bacterial numbers (Gerhardt, 1981; Dong *et al.*, 1999).

## Results

### *NbALY916* silencing inhibits cell death in response to Nep1_Mo_

**Fig. 1.**
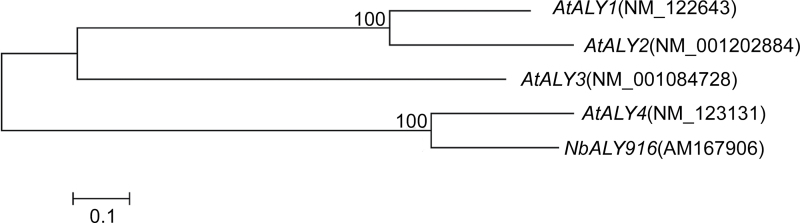
Phylogeny produced from the ALY sequence data of *Arabidopsis* and *N. benthamiana*. A bootstrap value of 100 supported the closest relationship between *NbALY916* and At*ALY4*.

*N. benthamiana* is the most widely used host for VIGS (Goodin *et al.*, 2008). Purified Nep1_Mo_ inoculated into *N. benthamiana* leaves can induce cell death (Zhang *et al*., 2012a). To identify components of the Nep1_Mo_ signalling pathway, a high-throughput *in planta* down-regulated expression screen of 6000 *N. benthamiana* cDNAs was performed using a PVX-based VIGS system (Supplementary Fig. S1 at *JXB* online). Each clone was used to infect two *N. benthamiana* plants. A common positive control is silencing of the *phytoene desaturase* (*PDS*) gene, which results in photobleaching of the silenced regions and is a readily visible phenotype. When photobleaching was apparent, two leaves of candidate plants were infiltrated with Nep1_Mo_ solution. Several cDNAs, including PVX clone #14-4-4, were found to compromise Nep1_Mo_-triggered cell death upon silencing. Since the silencing of #14-4-4 produced the strongest and most reproducible inhibition of cell death triggered by Nep1_Mo_, the #14-4-4-silenced line was therefore chosen for further study. The DNA sequence of #14-4-4 showed strong similarity to the C-terminal domain of ALY916 (GenBank accession no. AM167906) from *N. benthamiana*. To facilitate a more comprehensive comparative analysis of *NbALY916*, the full-length cDNA for *NbALY916* was isolated from *N. benthamiana*. Three clones of the full-length cDNA, named *NbALY916*, were sequenced to confirm that the correct gene had been cloned. The predicted protein for NbALY916 is composed of 275 amino acids. The most closely related protein is AtALY4 from *A. thaliana* (56% amino acid sequence similarity; Fig. 1).

### *NbALY916* silencing and the mutation of At*ALY4* reduces cell death and H_2_O_2_ accumulation following Nep1_Mo_ exposure

**Fig. 2.**
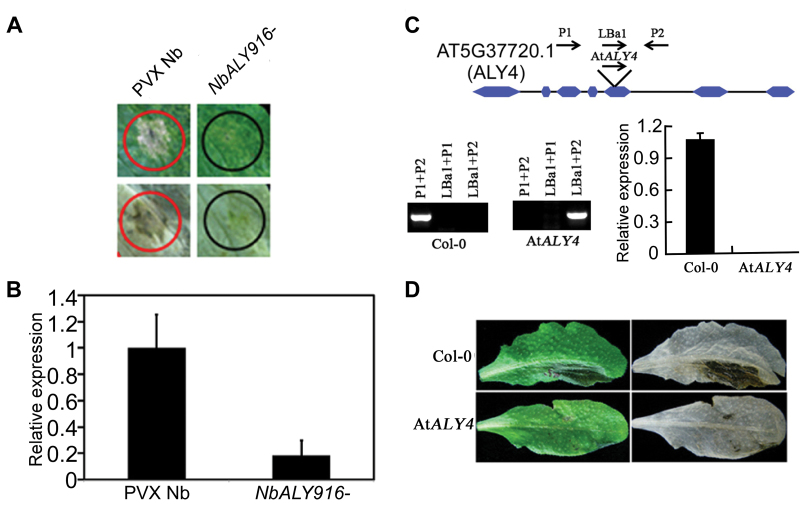
Silencing of *NbALY916*, the mutation in *Arabidopsis* At*ALY4*, and induction of hypersensitivity responses with Nep1_Mo_ (50nM). (A) Leaves (representative of three replicate treatments) from control PVX- and *NbALY916*-silenced *N. benthamiana* were infiltrated with Nep1_Mo_ solution simultaneously. The red and black circles indicate cell death and no cell death, respectively. Leaves were removed from plants after 2 d of treatment (upper row) and bleached in ethanol (lower row). (B) First-strand cDNA was generated from total RNA obtained from PVX-only plants or from plants silenced for NbALY916. qRT–PCR was performed with the cDNA and specific primers to the targeted gene, and *EF1α* as an endogenous control. (C) Schematic representation of T-DNA insertion sites in *AtALY4* and genomic structure of the Arabidopsis *AtALY4* gene. Exons and introns are represented as a blue box and black lines, respectively; PCR verification of the At*ALY4* T-DNA mutant. qRT-PCR analysis of AtALY4 and *AtEF1*α transcripts in the wild-type (Col-0) and At*ALY4* mutant. (D) Cell death phenotype on control and At*ALY4* mutant leaves after inoculation with Nep1_Mo_. Leaves were removed from plants after 2 d of treatment (upper row) and bleached in ethanol (lower row).

Reduced expression of *NbALY916* resulted in a strong phenotype, inhibiting cell death in *N. benthamiana* leaves after inoculation with Nep1_Mo_ (Fig. 2A). To confirm the suppression of *NbALY916* mRNA in the silenced plants, qRT–PCR was performed. A clear reduction in *NbALY916* mRNAs was observed in silenced plants compared with control plants infected by the PVX-VIGS vector alone (Fig. 2B; Supplementary Fig. S2 at *JXB* online). Thomas *et al.* (2001) reported that at least 23 nucleotides of perfectly matched sequence are necessary to target a gene for silencing (Thomas *et al.*, 2001). BLAST analysis revealed that NbALY916 showed no significant homology with any expressed sequence tags in the *N. benthamiana* database (http://compbio.dfci.harvard.edu/tgi/cgi-bin/tgi), and that it shared 100% identity with stretches of only 10 or fewer nucleotides in *NbALY617*, *NbALY1693*, and *NbALY615* (three homologues of *NbALY916* in *N. benthamiana*). To test whether the VIGS of *NbALY916* could affect the expression of the other *NbALY* genes, their transcriptional level was examined by qRT–PCR using primers that specifically annealed to these gene sequences. The results revealed that the expression of these genes was unaffected in the *NbALY916*-silenced plants (Supplementary Fig. S3). These results indicate a high degree of target specificity using VIGS. It was also observed that the Nep1_Mo_-triggered HR was not suppressed in *NbALY617*-, *NbALY1693*-, and *NbALY615*-silenced plants. These data demonstrate that the observed inhibition of cell death was due to the specific silencing of *NbALY916*.

As described above, *NbALY916* is closely related to an *A. thaliana* gene, *AtALY4*. To determine whether mutations in the *AtALY4* gene affect Nep1_Mo_-induced cell death, homozygous lines containing T-DNA insertions in this gene were obtained from the Salk Institute Genomic Analysis Laboratory collection by PCR screen. The T-DNA in the At*ALY4* mutant is inserted into the fifth exon of *AtALY4*. In the allele, wild-type *AtALY4* transcripts were did not detected by qRT–PCR (Fig. 2C; Supplementary S2 at *JXB* online). As expected, Col-0 seedlings showed obvious cell death after treatment with Nep1_Mo_. Conversely, the At*ALY4* mutant showed hyposensitivity to Nep1_Mo_ by displaying no cell death (Fig. 2D).

**Fig. 3.**
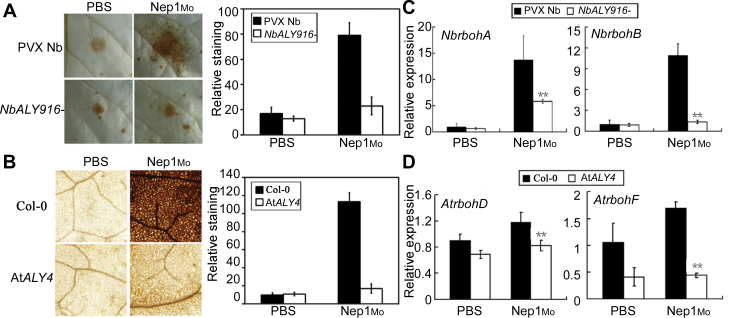
*In situ* detection of hydrogen peroxide using DAB staining on leaves of *NbALY916-*silenced *N. benthamiana* and the *Arabidopsis* At*ALY4* mutant in response to Nep1_Mo_. (A) *Nicotiana benthamiana* leaves silenced for *NbALY916* were compared with PVX-only leaves 6h after infiltration of PBS (10mM) or Nep1_Mo_ (50nM). Elicitation with the elicitor was conducted on plants by infiltrating an equivalent elicitor solution of 25 μl. Quantitative scoring of staining in leaves of the control and gene-silenced plants with Nep1_Mo_ treatment. The analysis was repeated for three sets of independently silenced plants in each experiment; the values shown were the means ±SD of duplicate assays. The experiment was repeated twice with similar results. (B) DAB staining of ROS accumulation in the *Arabidopsis* At*ALY4* mutant leaves 6h after inoculation with Nep1_Mo_. (C) *NbrbohA* and *NbrbohB* were analysed by qRT–PCR and normalized to *NbEF1α* expression. (D) *AtrbohD* and *AtrbohF* were analysed by qRT–PCR and normalized to *AtEF1α* expression. Each measurement is an average of three replicates; Experiments were repeated twice with similar results.

To test whether the alteration in Nep1_Mo_-induced cell death was associated with H_2_O_2_ accumulation, the H_2_O_2_ levels were quantified in vector control and *NbALY916*-silenced plants. H_2_O_2_ production in response to Nep1_Mo_ was much lower in *NbALY916*-silenced plants (75% reduction over control treatment; Fig. 3A). Light staining was also observed on At*ALY4* mutant leaves following Nep1_Mo_ treatment (Fig. 3B). *N. benthamiana rbohA* and *rbohB* and *A. thaliana rbohD* and *rbohF*, which encode plant NADPH oxidases, are responsible for generating the H_2_O_2_ involved in HRs and plant resistance (Torres *et al.*, 2002; Yoshioka *et al.*, 2003; Sagi and Fluhr, 2006). The expression level of *rboh* genes (*NbrbohA* and *NbrbohB*, and *AtrbohD* and *AtrbohF*) was decreased significantly in *NbALY916*-silenced and At*ALY4* mutant lines compared with control plants (Fig. 3C, D; Supplementary Fig. S4 at *JXB* online). These data demonstrate that ALYs regulate H_2_O_2_ accumulation in response to Nep1_Mo_. These results suggest that the compromised cell death observed in *NbALY916*-silenced plants and the At*ALY4* mutant may be associated with a Nep1_Mo_-induced decrease in H_2_O_2_.

### *NbALY916* silencing and the mutation of *AtALY4* impairs Nep1_Mo_-activated stomatal closure

**Fig. 4.**
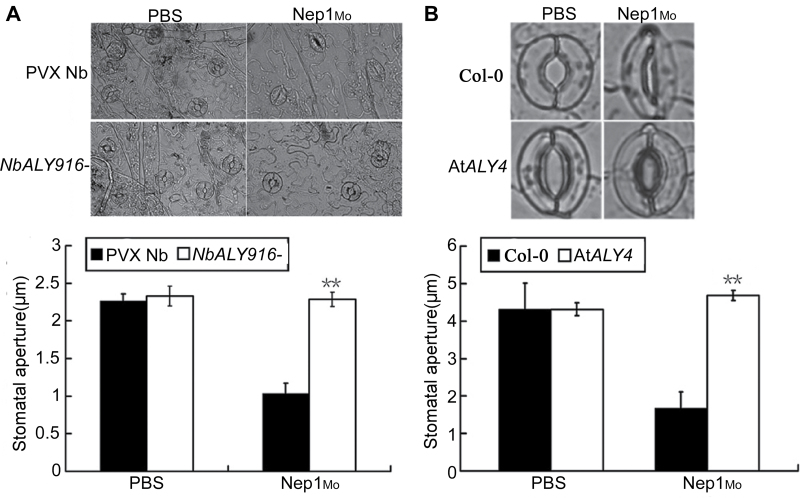
Stomatal aperture measurements show that Nep1_Mo_-induced stomatal closure is reduced in *NbALY916*-silenced *N. benthamiana* and the *Arabidopsis* At*ALY4* mutant. Stomatal aperture of *N. benthamiana* (A) and *Arabidopsis* (B) was measured 3h after incubation in PBS (10mM) and Nep1_Mo_ (50nM). Values represent means ±SE from three independent experiments; *n*=50 apertures per experiment. Data were compared using the Student’s *t*-test at the 95% significance level.

Recent studies have shown that stomata play an active role in the innate immune system (Melotto *et al.*, 2006, 2008). It has been reported that elicitors trigger *RBOH*-, *VPE*-, and G protein-dependent stomatal closure in *N. benthamiana* leaves (Zhang *et al.*, 2009, 2010, 2012a), but whether ALY proteins contribute to elicitor-induced stomatal closure remains unclear. The stomatal response of *NbALY916*-silenced plants exposed to Nep1_Mo_ was examined. It was found that *NbALY916*-silenced plants failed to close their stomata in response to Nep1_Mo_, whereas the stomata of control plants showed normal closure responses (Fig. 4A). These results suggest that *NbALY916* silencing in plants affects the response to Nep1_Mo_ or affects a general step in stomatal closure that is common to multiple stomatal closure pathways. The At*ALY4* mutant was also tested for Nep1_Mo_-induced stomatal closure. At*ALY4* mutant plants were defective in Nep1_Mo_-induced stomatal closure (Fig. 4B). Nep1_Mo_-induced stomatal aperture analyses were consequently performed on the *NbALY916*-silenced plants and At*ALY4* mutants. It was found that the *NbALY916*-silenced plants and At*ALY4* mutant plants showed a markedly reduced response to Nep1_Mo_. Therefore, *N. benthamiana NbALY916* and *A. thaliana AtALY4* are involved in the Nep1_Mo_ signalling pathways leading to stomatal closure.

### *NbALY916* silencing and the mutation of *AtALY4* decreases Nep1_Mo_-mediated NO production in guard cells

**Fig. 5.**
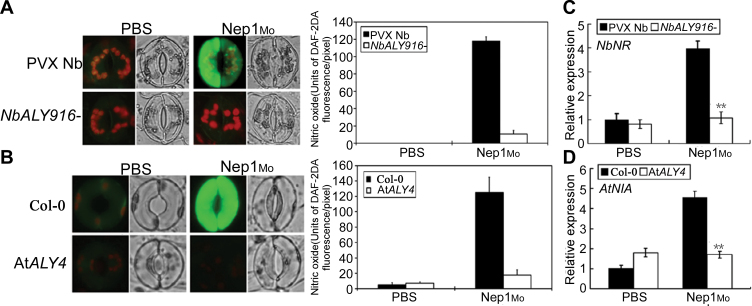
Effects of silencing of *NbALY916* and mutation in *Arabidopsis* At*ALY4* on NO burst in guard cells in response to Nep1_Mo_. (A) In all cases, the NO-sensitive dye DAF-2DA was loaded into cells of the epidermal peels, and fluorescence was measured after addition of PBS (10mM) and Nep1_Mo_ (50nM). Quantitative analysis of *in vivo* NO generation monitored using DAF-2DA fluorescence as shown in A. (B) The epidermal peels of the At*ALY4* mutant stained with DAF-2DA upon Nep1_Mo_ treatment and quantification of NO accumulation shown in B. For each treatment, fluorescence and bright-field images were shown. Results from several experiments were compiled in this figure. Experiments were repeated at least three times, and representative images are shown in A. Green indicates the NO burst. Results were presented as the mean (*n* ≥3) fluorescence intensity per pixel. (C) *NbNR* was analysed by qRT–PCR and normalized to *NbEF1α* expression. (D) *AtNIA* was analysed by qRT–PCR and normalized to *AtEF1α* expression. Each measurement is an average of three replicates; experiments were repeated twice with similar results.

NO functions as a signal in plant disease resistance (Delledonne *et al.*, 1998; Desikan *et al.*, 2002). It was previously shown that NO plays a critical role in Nep1_Mo_-induced stomatal closure (Zhang *et al.*, 2012*b*). The *NbALY916*-silenced plants and At*ALY4* mutants were insensitive to Nep1_Mo_-induced stomatal closure (Fig. 4). To determine further the cellular role of ALYs in the regulation of stomatal closure, NO production was compared in *NbALY916*-silenced and control plants using the NO-specific fluorescent dye DAF-2DA. Without Nep1_Mo_ treatment, the guard cells of the control and *NbALY916*-silenced plants exhibited similar basal staining for NO. The treatment of epidermal strips with Nep1_Mo_ induced a rapid increase in NO levels, as indicated by the change in fluorescence intensity compared with control (PBS) treatment. An ~90% increase in NO occurred in the guard cells of control plants following Nep1_Mo_ treatment. However, no increase in NO was observed in the guard cells of *NbALY916*-silenced plants after Nep1_Mo_ treatment (Fig. 5A). Similarly, a decrease in Nep1_Mo_-induced NO was observed in the At*ALY4* mutant (Fig. 5B). The expression levels of nitrate reductase (*N. benthamiana* NR, *A. thaliana* NIA) also decreased sharply in the *NbALY916*-silenced and At*ALY4* mutant lines compared with control plants (Fig. 5C, D; Supplementary Fig. S4 at *JXB* online). This suggests that NR may be the main resource of NO production in Nep1_Mo_ signalling. Taken together, these results indicate that ALY proteins are required for Nep1_Mo_-mediated NO production in guard cells.

### NO acts downstream of ALY and H_2_O_2_

In addition to NO, H_2_O_2_ is also involved in elicitor-induced stomatal closure (Srivastava *et al.*, 2009). To investigate the possible interaction between ALY, H_2_O_2_, and NO in Nep1_Mo_-induced stomatal closure, the effects of an NO scavenger (cPTIO) on Nep1_Mo_-, H_2_O_2_-, and NO-induced stomatal closure were assessed in *NbALY916*-silenced plants or At*ALY4* mutant lines. Nep1_Mo_-induced stomatal closure was greatly reduced in the presence of cPTIO, in agreement with previous reports (Zhang *et al.*, 2012*b*). Here, cPTIO inhibition of SNP-induced stomatal closure was observed, similar to the inhibition of Nep1_Mo_-induced stomatal closure (*P*<0.001), indicating a requirement for NO in Nep1_Mo_-induced stomatal closure and that NO does not require H_2_O_2_ generation to initiate stomatal closure.

**Fig. 6.**
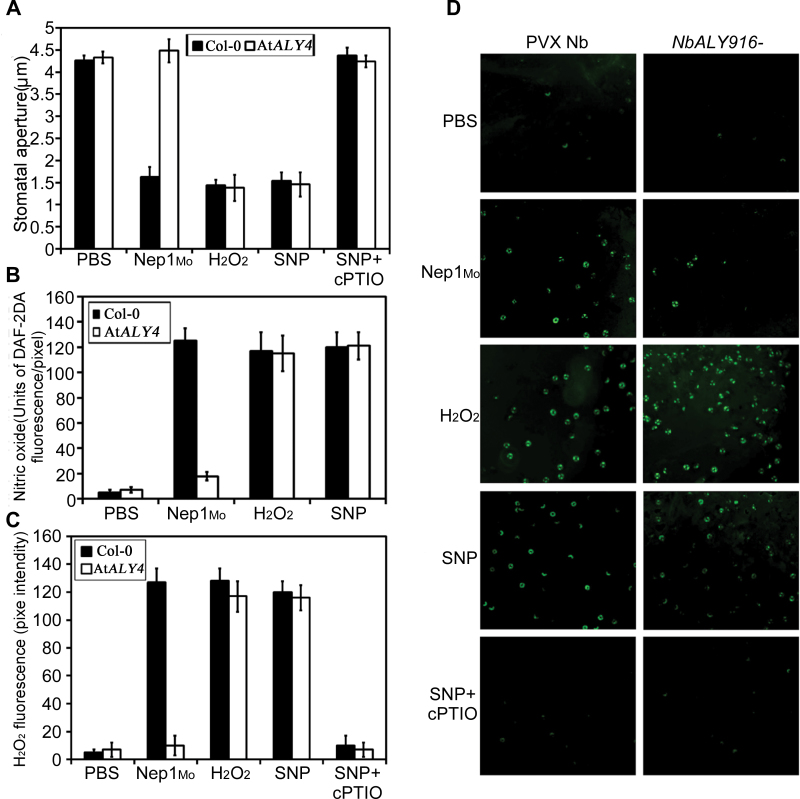
NO generation is required for H_2_O_2_-induced stomatal closure. (A) Wild-type *Arabidopsis* and At*ALY4* mutant leaves were treated with Nep1_Mo_, H_2_O_2_, or SNP in the absence or presence of 2-phenyl-4,4,5,5-tetremethylimidazolinone-1-oxyl 3-oxide (cPTIO). Stomatal apertures were measured 3h after treatment. The data are displayed as estimated means and associated 95% confidence intervals (CIs). (B) Epidermal fragments were incubated with DAF2-DA in MES-KCl buffer. NO synthesis was monitored in controls and 3h after treatment with Nep1_Mo_, H_2_O_2_, or SNP, in the absence or presence of cPTIO. Data are displayed as estimated mean pixel intensities and associated 95% CIs. (C) Quantitative analysis of *in vivo* H_2_O_2_ generation monitored using H_2_DCF fluorescence as shown in D. (D) Epidermal fragments were incubated with (H_2_DCFDA) in MES-KCl buffer. Hydrogen peroxide synthesis was monitored in controls and 3h after treatment with Nep1_Mo_, H_2_O_2_, or SNP, in the absence or in the presence of cPTIO.

The role of NO in H_2_O_2_-induced stomatal closure was further examined by monitoring NO synthesis in response to applied H_2_O_2_. Epidermal fragments were loaded with DAF-2DA to test for changes in NO-induced fluorescence. A significant increase in NO-induced guard cell fluorescence was observed in H_2_O_2_-treated epidermal fragments compared with control tissue (*P*<0.001), demonstrating H_2_O_2_-mediated NO production in guard cells (Fig. 6B). Importantly, SNP-induced NO synthesis was abolished by co-incubation with cPTIO, correlating these data with those from the stomatal bioassays (*P*<0.001; Fig. 6A). Moreover, NO-induced fluorescence was not significantly different between Nep1_Mo_- and H_2_O_2_-treated epidermal fragments (*P*>0.05).

The data in Fig. 6A indicate that H_2_O_2_ is not required for NO-induced stomatal closure. However, He *et al.* (2013) reported that in *Vicia faba* guard cells, exogenous NO, applied in the form of the NO donor SNP, did induce H_2_O_2_ production. Consequently, the effects of SNP on H_2_O_2_ generation in *A. thaliana* guard cells were analysed using the fluorescent probe H_2_DCFDA (Fig. 6C). SNP did induce guard cell H_2_DCF fluorescence, as did Nep1_Mo_ treatment, which has been previously shown to mediate H_2_O_2_ production (Pei *et al.*, 2000; Desikan *et al.*, 2002). However, H_2_DCFDA is not specific for H_2_O_2_ and it also reacts with NO (Hempel *et al.*, 1999). To determine whether the effects of SNP treatment on H_2_DCF fluorescence were merely attributable to the reaction of NO with H_2_DCF, a number of experimental treatments were performed (Fig. 6D). Most importantly, treatment with cPTIO greatly reduced H_2_DCF fluorescence in response to SNP. The reaction with cPTIO is specific to NO and not H_2_O_2_; therefore, these data indicate that the fluorescence observed in the SNP-treated guard cells was due to exogenously applied NO and not H_2_O_2_, and NO does not induce H_2_O_2_ production in *N. benthamiana* or *A. thaliana*. Together, these data demonstrate unequivocally that NO functioned downstream of H_2_O_2_ and was involved in ALY-mediated stomatal closure triggered by Nep1_Mo_.

### *NbALY916* participates in the Nep1_Mo_-triggered resistance of *N. benthamiana* to *P. nicotianae*

**Fig. 7.**
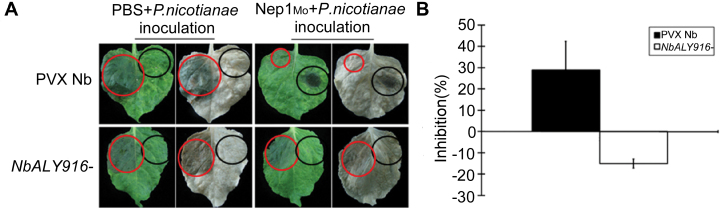
*NbALY916*-silenced plants display enhanced sensitivity to *P. nicotianae*. (A) For the control and gene-silenced plants, fully expanded leaves collected 4h after Nep1_Mo_ treatment (10 μl, black circle) were inoculated with a 2 mm×2mm hyphal plug on the left leaf surfaces opposite the Nep1_Mo_-infiltrated sides. Then, the leaves were placed in Petri dishes containing filter paper saturated with sterilized distilled water and kept under a 16h day/8h night regime at 25 °C. Pictures of the lesions were taken at 48h post-inoculation and the lesion diameter (red circle) was measured. (B) Resistance evaluation based on diameter of lesion spots. Inhibition rate=(diameter of necrosis with PBS treatment–diameter of necrosis with Nep1_Mo_ treatment)/diameter of control. Data are means ±SE from eight experiments.

Elicitors can induce systemic resistance in plants (Garcia-Brugger *et al.*, 2006; Yang *et al.*, 2009). Here it was tested whether the silencing of *NbALY916* had effects on Nep1_Mo_-triggered disease resistance against *P. nicotianae.* All of the *NbALY916*-silenced leaves were infiltrated with Nep1_Mo_ at one spot, and 4h later the leaf surfaces opposite the Nep1_Mo_-infiltrated sides were inoculated with 2 mm×2mm mycelial plugs of *P. nicotianae*. Systemic resistance was assessed 48h after *P. nicotianae* inoculation by comparing and measuring the sizes of the lesions. Typical water-soaked *Phytophthora* lesions appeared within 24h post-inoculation (hpi). Expanding disease lesions around the inoculated spots were observed in the silenced plants at 48 hpi. However, Nep1_Mo_ treatment significantly inhibited the expansion of lesions in PVX control-inoculated leaves of *N. benthamiana* (Fig. 7). These findings indicate that *NbALY916* is required for Nep1_Mo_-induced disease resistance to *P. nicotianae*.

### *AtALY4* is involved in Nep1_Mo_-triggered resistance towards *Pst* DC3000

**Fig. 8.**
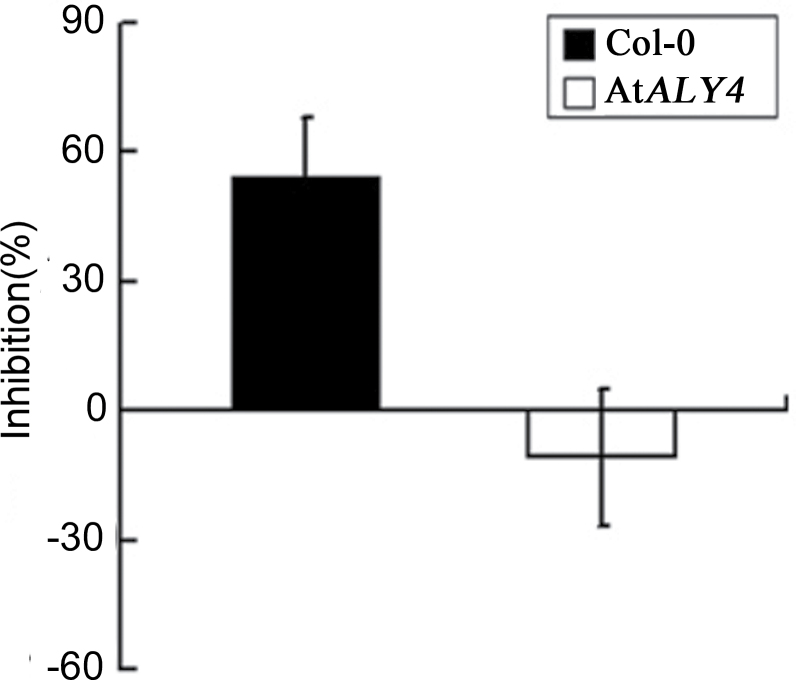
Nep1_Mo_ induced a significant increase in number and size of *Pst* DC3000 lesions on At*ALY4* mutant lines. Resistance evaluation based on diameter of *Pst* DC3000 lesion spots.

To determine whether *AtALY4* has an effect on *Pst* DC3000-induced disease symptoms upon Nep1_Mo_ treatment in *A. thaliana*, leaves from At*ALY4* mutant plants and Col-0 were infiltrated with Nep1_Mo_ at one spot, then wild-type and At*ALY4* mutant plants were infiltrated (10^8^ cfu ml^–1^) with *Pst* DC3000 using a syringe. As expected, Col-0 showed water-soaked necrotic lesions accompanied by chlorosis, while the At*ALY4* mutant plants exhibited accelerated necrotic lesions without visible chlorosis upon PBS treatment. Interestingly, when the growth of *Pst* DC3000 was monitored at 0, 1, 2, and 4 d post-infiltration, the bacterial population on the At*ALY4* mutant was significantly increased compared with that on the inoculated control plants in response to Nep1_Mo_ (Fig. 8). These results suggest that *AtALY4* significantly contributed to Nep1_Mo_-induced plant resistance against *Pst* DC3000 in *A. thaliana*, at least for the length of time that bacterial growth was monitored.

### *NbALY916*-silenced and At*ALY4* mutant plants show altered defence-related gene expression

**Fig. 9.**
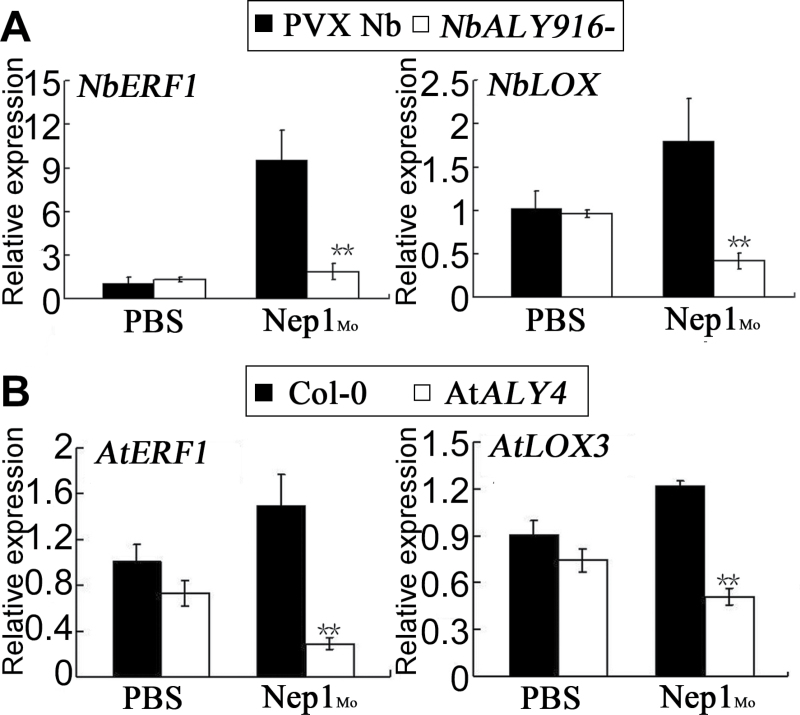
Expression analysis of *ERF* and *LOX* in response to Nep1_Mo_ (50nM). (A) At 6h after treatment with or without Nep1_Mo_ (50nM), leaf samples were harvested from the inoculation site on the lower and the upper leaves; *N. benthamiana EF1α* expression is used to normalize the expression value in each sample, and relative expression values were determined against buffer or PVX-infected plants using the comparative Ct method (2^–ΔΔCt^). (B) Transcript levels of *ERF* and *LOX* in the wild-type and the *Arabidopsis* At*ALY4* mutant 6h after inoculation with Nep1_Mo_. Bars represent the mean (three biological replicates) ±SD.

Differences in the accumulation of H_2_O_2_ and NO were detected between the *NbALY916*-silenced plants and control plants. Distinct levels existed in the At*ALY4* mutant and wild type as well, suggesting that the *NbALY916*-silenced plants and At*ALY4* mutant exhibit altered defence-related gene expression. To address this possibility, the kinetics of the expression of selected genes following Nep1_Mo_ inﬁltration were examined by qRT–PCR not only in *NbALY916*-silenced and PVX control plants, but also in At*ALY4* mutant and wild-type plants (Fig. 9; Supplementary Fig. S4 at *JXB* online). The jasmonic acid signalling gene *LOX* encodes a lipoxygenase (Delker *et al.*, 2006), while *ERF1* is involved in ethylene signalling (Yang *et al.*, 1997; Alonso *et al.*, 1999; Ding *et al.*, 2002). The expression of these genes showed no significant difference in *NbALY916*-silenced plants and control plants treated with PBS. After treatment with Nep1_Mo_, the expression levels of *ERF1* and *LOX3* were up-regulated by 9- and 1.8-fold compared witho their previous expression levels, respectively. However, the Nep1_Mo_-mediated expression of *ERF1* and *LOX3* was down-regulated by 7- and 5-fold, respectively, in the *NbALY916*-silenced plants compared with control plants. Such suppression of the Nep1_Mo_-mediated expression of *ERF1* and *LOX3* was also detected in the At*ALY4* mutant compared with the wild type. These results reveal that the silencing of *NbALY916* in *N. benthamiana* and the mutation of *AtALY4* both influence the expression of defence-related genes.

## Discussion

**Fig. 10.**
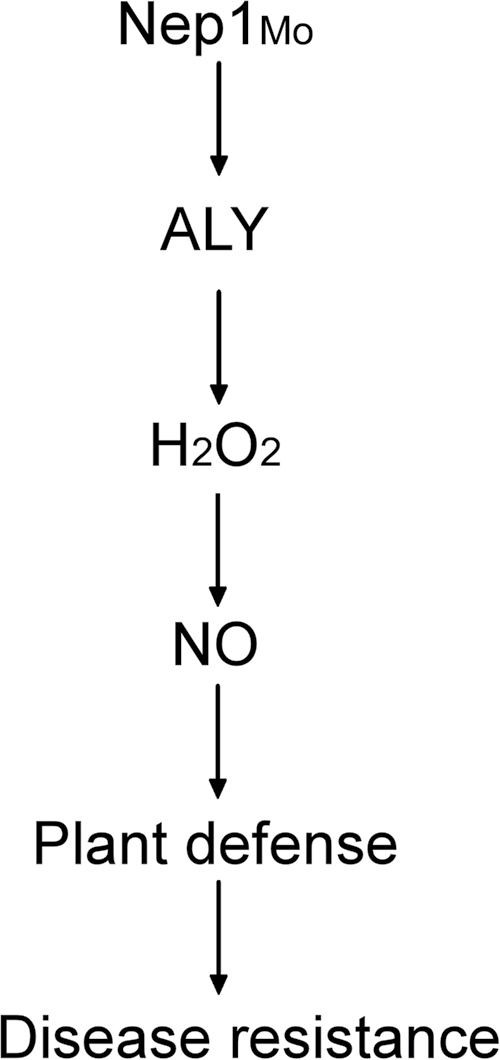
Speculative model of new signalling components in the Nep1_Mo_-activated signalling pathway. Virus-induced gene silencing of *NbALY916* and mutation of *Arabidopsis* At*ALY4* placed *ALY* downstream of the Nep1_Mo_ recognition event. NO acts downstream of ALY and H_2_O_2_ in guard cells to contribute to Nep1_Mo_-induced resistance.

The observation that Nep1_Mo_ can induce cell death in *N. benthamiana* and the ability to conduct VIGS in *N. benthamiana* provided an excellent strategy for identifying plant genes that play a role in Nep1_Mo_ signalling. Here, it was found that a loss of the *N. benthamiana* gene *NbALY916* and its orthologue (*AtALY4*) in *A. thaliana* resulted in the inhibition of Nep1_Mo_-mediated cell death and stomatal closure. The inoculation of *NbALY916*-silenced *N. benthamiana* with *P. nicotianae* induced accelerated necrotic lesions upon Nep1_Mo_ treatment. Similar to *NbALY916*-silenced *N. benthamiana*, inoculation with *Pst* DC3000 of the At*ALY4* mutant line induced accelerated necrotic lesions in response to Nep1_Mo_. Moreover, it was found that NO acts downstream of ALY and H_2_O_2_ to participate in Nep1_Mo_ signalling. Based on the phenotypes observed, a model was developed that involves ALY, H_2_O_2_, and NO in Nep1_Mo_-mediated immunity (Fig. 10).

### *NbALY916* silencing and the mutation of At*ALY4* suppresses Nep1_Mo_-triggered HR and stomatal closure

ALY proteins, which belong to a highly conserved polypeptide nuclear localization signal protein family, exist in plants, yeast, *Drosophila*, nematodes, and mammals. A previous study found that ALY proteins play an important role in the activation of transcription, pre-mRNA splicing, and mRNA export in mammals (Zhou *et al.*, 2000; Gatfield and Izaurralde, 2002). However, no subsequent reports were found regarding the function of ALY proteins in plants. Unlike previous strategies, VIGS was employed to investigate the role of *NbALY916* in Nep1_Mo_-mediated HR. The results indicate that the silencing of *NbALY916* and mutation of its orthologue in *A. thaliana*, AtALY4, compromised Nep1_Mo_-mediated HR, confirming a positive role for ALYs in cell death regulation. ALY-mediated cell death caused by Nep1_Mo_ was associated with the rapid generation of H_2_O_2_, displaying a similarity to pathogen-induced HRs. Most importantly, an ALY loss-of-function study demonstrated that ALY is involved in the regulation of HRs caused by the *M. oryzae* elicitor Nep1_Mo_. These results indicate that ALYs may be a convergence point for Nep1_Mo_ signal transduction pathways.

The present results show that the silencing of *NbALY916* and mutation of *AtALY4* compromised Nep1_Mo_-induced stomatal closure and was accompanied by less NO accumulation in guard cells (Fig. 4 and 5), which suggests that ALYs control Nep1_Mo_-mediated stomatal closure. NO is a key mediator of abscisic acid (ABA)-induced stomatal closure in peas (Neill *et al.*, 2002), *V. faba* (Garcia-Mata and Lamattina, 2002, 2003), and *A. thaliana* (Bright *et al.*, 2006). These data indicate that ALY proteins mediate Nep1_Mo_-triggered stomatal closure via NO signalling. An increasing number of studies in plants have confirmed the importance of stomata in plant immunity, and stomatal closure has been observed as a result of PTI. The results showed that Nep1_Mo_-induced resistance against *P. nicotianae* was also compromised in *NbALY916*-silenced plants (Fig. 7). This suggests that *NbALY916* is involved in the regulation of plant immunity, controlling stomatal apertures and inhibiting the entry of pathogens into plant leaves during infection.

### NO acts downstream of ALY-mediated H_2_O_2_ generation in response to Nep1_Mo_

The requirement for NO and H_2_O_2_ in elicitor-mediated stomatal closure has been shown previously (Zhang *et al.*, 2009). However, NO and H_2_O_2_ synthesis were thought to occur in parallel, until Lum *et al.* (2002) demonstrated that exogenous H_2_O_2_ induced the rapid production of NO in *Phaseolus aureus* guard cells. A report provided pharmacological evidence indicating that endogenous H_2_O_2_-mediated NO generation plays an important role in UV-B-induced stomatal closure in *V. faba* (He *et al.*, 2013). In the present study, NO synthesis in response to H_2_O_2_ in *N. benthamiana* and *A. thaliana* guard cells was demonstrated. Importantly, this process was correlated with Nep1_Mo_-induced stomatal closure. Using a pharmacological approach, it has been shown that NO synthesis is required for H_2_O_2_-induced stomatal closure. In contrast to the findings of He *et al.* (2013), the data in the present study demonstrate that NO does not induce H_2_O_2_ synthesis as required for stomatal closure. Moreover, the data show unequivocally that NO does not induce H_2_O_2_ synthesis in guard cells. Rather, the H_2_DCF fluorescence monitored in the presence of SNP was actually attributable to the reaction of the dye with NO, not H_2_O_2_. However, He *et al.* (2013) did report that SNP induced H_2_DCF fluorescence and stomatal closure, so it is possible that guard cell responses differ between *N. benthamiana*, *A. thaliana*, and *V. faba*.

Further evaluation of this response and the potential source of NO induced by exogenous H_2_O_2_ revealed that NO production and stomatal closure could both be attributed to NR activity. The expression level of NR in *NbALY916*-silenced plants and At*ALY4* mutants in Nep1_Mo_-induced NO synthesis and stomatal closure was significant and indicates a role for NR activity in these responses (Fig. 5C, D). Previous studies have reported that H_2_O_2_-induced NO generation in the guard cells of *V. faba* was related to NR activity. The analysis of NR–NO activity provides unequivocal evidence that *A. thaliana* NR has the capacity to generate NO from nitrite (Bright *et al.*, 2006). Guard cells of the double mutant *nia1, nia2* do not generate NO in response to H_2_O_2_, indicating that NR is responsible for the production of NO in guard cells in response to exogenous H_2_O_2_. Importantly, it has been demonstrated that *nia1, nia2* plants do generate H_2_O_2_ in response to ABA, providing further evidence that NR acts downstream of ABA-mediated H_2_O_2_ generation to produce NO and to induce stomatal closure. It has been suggested that NR-mediated NO generation may act downstream of ALY-mediated H_2_O_2_ generation in Nep1_Mo_ signalling.

### *NbALY916* silencing and the mutation of At*ALY4* suppresses Nep1_Mo_-induced pathogen resistance

*NbALY916*-silenced plants and At*ALY4* mutants exhibited increased susceptibility to pathogen invasion. The data presented suggest that ALYs function as a positive regulator of broad spectrum resistance during pathogen invasion. The silencing of *NbALY916* in *N. benthamiana* conferred decreased resistance to *P. nicotianae* after Nep1_Mo_ treatment. Moreover, upon Nep1_Mo_ application, *NbALY916*-silenced *N. benthamiana* showed a decrease in *LOX* and *ERF* expression compared with control plants, suggesting that *NbALY916* is critically involved in Nep1_Mo_-triggered defence responses. At*ALY4* plants homozygous for the T-DNA insertion were susceptible to infection by *Pst* DC3000 upon Nep1_Mo_ treatment. As expected, decreased *LOX* and *ERF* expression was observed in the At*ALY4* mutant relative to wild-type plants during *Pst* infection. Interestingly, this suggests that *AtALY4* functions in the *A. thaliana* defence response to *Pst* infection in a manner similar to that of *N. benthamiana* upon *P. nicotianae* infection. The decreased level of salicylic acid (SA)-responsive (*LOX*) and ethylene-responsive (*ERF*) gene expression in *N. benthamiana* and *A. thaliana* supports the notion that ALYs are involved in SA and ethylene-mediated signalling pathways during pathogen infection. Analyses of *NbALY916*-silenced and At*ALY4* mutant plants revealed that plant disease resistance is conferred by *ALY* gene expression during pathogen infection.

In conclusion, a VIGS-based forward genetic screen was developed for the identification of new targets involved in Nep1_Mo_ signalling. Although the original intention of the study was to identify genes involved in Nep1_Mo_-induced HR, a gene, *NbALY916*, that when silenced inhibited cell death and stomatal closure upon Nep1_Mo_ application was identified. Although the precise role of *NbALY916* in the Nep1_Mo_ signalling pathway is debatable and requires confirmation, the results present a new role for ALYs in *P. nicotianae* and bacterial disease development.

## Supplementary data

Supplementary data are available at *JXB* online.

Fig. S1 Screening of *Nicotiana benthamiana* gene coding hypersensitive cell death induced by Nep1_Mo_ by virus-induced gene silencing.

Fig. S2 Expression analysis of *NbALY916* and *AtALY4* in *NbALY916*-silenced plants and the At*ALY4* mutant by qRT–PCR.

Fig. S3 Local induction of hypersensitivity responses on PVX-, PVX.NbALY615-, PVX.NbALY617-, and PVX.NbALY1693-infected *N. benthamiana* leaves in response to Nep1_Mo_.

Fig. S4 Expression analysis of genes associated with redox control in *NbALY916*-silenced plants and the At*ALY4* mutant by qRT–PCR.

Table S1. Gene-specific primers for qRT–PCR.

Supplementary Data
