# Supplementary material for: ALY proteins participate in multifaceted Nep1Mo-triggered responses in Nicotiana benthamiana and Arabidopsis thaliana
Source: J Exp Bot. 2014 Apr 10;65(9):2483–94. doi: 10.1093/jxb/eru136 (PMC4036512; doi:10.1093/jxb/eru136)
Supplement: Supplementary Data [file supp_65_9_2483__index.html]

ALY proteins participate in multifaceted Nep1Mo-triggered responses in Nicotiana benthamiana and Arabidopsis thaliana — ALY proteins participate in multifaceted Nep1Mo-triggered responses in Nicotiana benthamiana and Arabidopsis thaliana — Supplementary Data 

# ALY proteins participate in multifaceted Nep1Mo-triggered responses in *Nicotiana benthamiana* and *Arabidopsis thaliana*

## Supplementary Data

Data files

**Files in this Data Supplement:**

- Supplementary Data - Supplementary Data
