# Supplementary material for: ALY proteins participate in multifaceted Nep1Mo-triggered responses in Nicotiana benthamiana and Arabidopsis thaliana
Source: J Exp Bot. 2014 Apr 10;65(9):2483–94. doi: 10.1093/jxb/eru136 (PMC4036512; doi:10.1093/jxb/eru136)
Supplement: Supplementary Data [file supp_eru136_jexbot122127_file001.pdf]

ALY proteins participate in multifaceted Nep1$_{Mo}$-triggered responses in *Nicotiana benthamiana* and *Arabidopsis*

*Wenjun Teng[#], Huajian Zhang[#], Wei Wang[#], Deqing Li, Meifang Wang, Jiewen Liu, Haifeng Zhang, Xiaobo Zheng, and Zhengguang Zhang**

Supplementary Material

Supplementary Table ST1

Gene-specific primers by qRT-PCR

Primer	qRT-PCR primers
NbALY916-F	GCCATCTGCCACCAGAATTGCC
NbALY916-R	CTTCCACGAGCTGAGCCAGGCAT
NbALY916-F1	TTCTGCGTCTGCTGCTAATC
NbALY916-R1	CTGACTTCTCAACGCCATTC
NbActin-F	ATGGCAGACGGTGAGGATATTCA
NbActin-R	GCCTTTGCAATCCACATCTGTTG
NbEF1α-F	GCTCTTAACGTCGGATGGTC
NbEF1α-R	AGCCAAACCCTAGCTCCATT
NbERF1-F	GCTCTTAACGTCGGATGGTC
NbERF1-R	AGCCAAACCCTAGCTCCATT
NbLOX-R	TCTAAGCTCATAAGCAATGG
NbLOX-F	CCTTAAGAGGAGATGGAACT
NbrbohA-F	AGCCAAACCCTAGCTCCATT
NbrbohA-R	GAGCTCTATGAGCGCTGGAA
NbrbohB-F	GTGATGCTCGTTCTGCTCTT
NbrbohB-R	CTTTAGCCTCAGGGTGGTTG
NbNR-F	GTGTGGCCCTAATTCCAAGA
NbNR-R	CGTCAATAACGGCACAGAGA
AtALY4-F	CCTTATATCTTCATTTGTCACA
AtALY4-R	CCTGTCCGTAGGGTCAGG
AtERF1-F	CCTTCCGATCAAATCCGTAA
AtERF1-R	GCAGCTTGATCGTAGGCTAA
AtLOX3-F	CAGACAGCTGAGTGTTCTTC
AtLOX3-R	GTTCTGCTGGTAATGCTTGG
AtRbohD-F	GAGAGCAGCCTCAACAACAC
AtRbohD-R	GTGGCAATGAACTTGAGACC
AtRbohF-F	CGGTGATCAAGAGTTCGTTG
AtRbohF-R	GCTCTTGTGAGAATTGTCGG
AtNIA-F	GCCATTATCCCACCATGAAC
AtNIA-R	GTTATGGCTCTCGTCTTCGT
AtS16-F	GGCGACTCAACCAGCTACTGA
AtS16-R	CGGTAACTCTTCTGGTAACGA
AtEF1α-F	ACCA CATGATTGAGAGGTCC
AtEF1α-R	GCATCTCAACAGACTTGACC

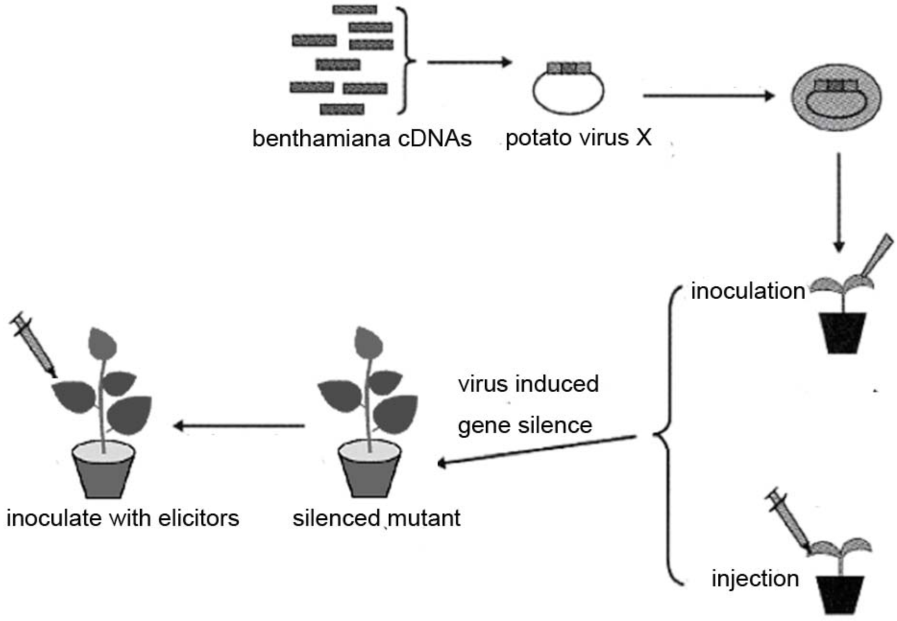

Fig. S1 Screening of *Nicotiana benthamiana* gene coding hypersensitive cell death induced by $Nep1_{Mo}$ by virus-induced gene silencing. cDNAs of *N.benthamiana* was cloned into PVX (pGR107) to generate PVX.cDNA. The construction was transferred into *Agrobacterium tumefaciens* strain GV3101. The bacteria suspension was inoculated into the underside of plant leaves. Silenced plant was acquired 4-5 weeks after inoculation. Leaves from control PVX and silenced *N.benthamiana* were infiltrated with the $Nep1_{Mo}$ simultaneously.

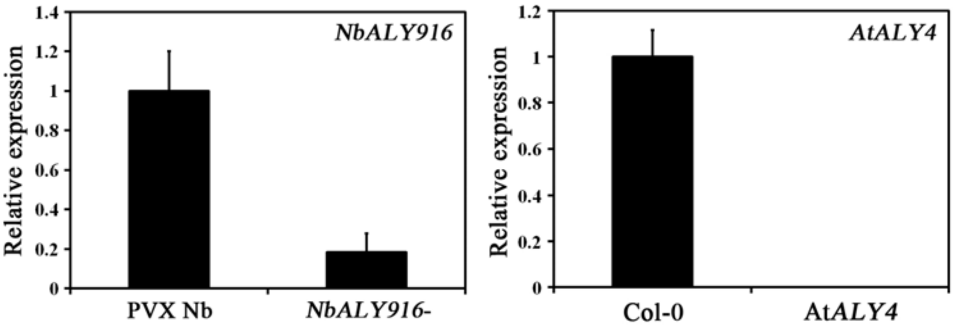

Figure S2. Expression analysis of *NbALY916* and *AtALY4* in *NbALY916*-silenced plants and At*ALY4* mutant by qRT-PCR. First-strand cDNA was generated from total RNA obtained from PVX-only plants or from plants silenced for *NbALY916*. qRT-PCR was performed with the cDNA and specific primers to the targeted gene and *Actin* as an endogenous control. qRT-PCR analysis of *AtALY4* and *AtS16* transcripts in the wild-type (Col-0) and At*ALY4* mutant.

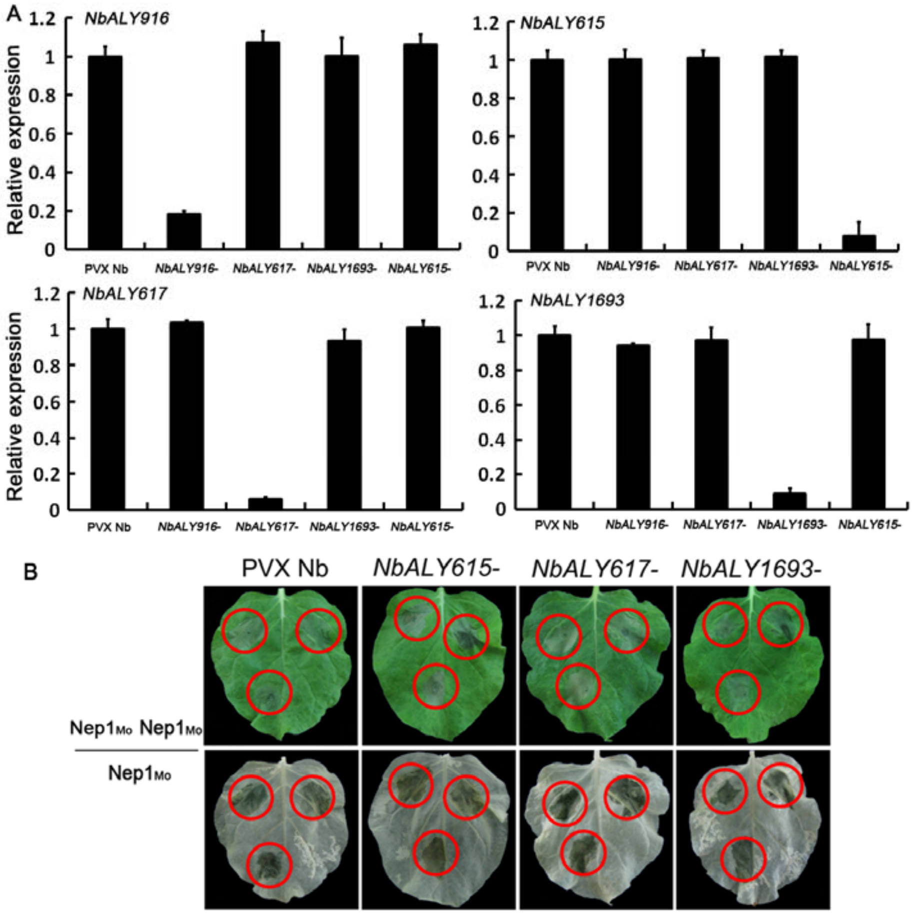

Figure S3. Local induction of hypersensitivity responses on PVX, PVX. NbALY615, PVX. NbALY617, and PVX. NbALY1693-infected *N. benthamiana* leaves in response to $Nep1_{Mo}$.

(A) PVX.NbALY615, PVX.NbALY617, PVX.NbALY1693, and PVX.NbALY916 specifically silence their respective target genes. Relative expression of *NbALY615*, *NbALY617*, *NbALY1693*, and *NbALY916* determined by qRT-PCR in *N. benthamiana* plants 3 weeks after *Agrobacterium* inoculation. RNA was extracted from leaves infected with PVX empty vector or the gene-specific PVX constructs. The error bars were derived from three technical replicates. (B) Leaves (representative of three replicate treatments) from control PVX, *NbALY615*-, *NbALY617*-, and *NbALY1693*-silenced *N. benthamiana* were infiltrated with the $Nep1_{Mo}$ *simultaneously*. The red circles indicate cell death. Leaves were removed from plants after 3 days of treatment (upper panels) and bleached in ethanol (lower panels).

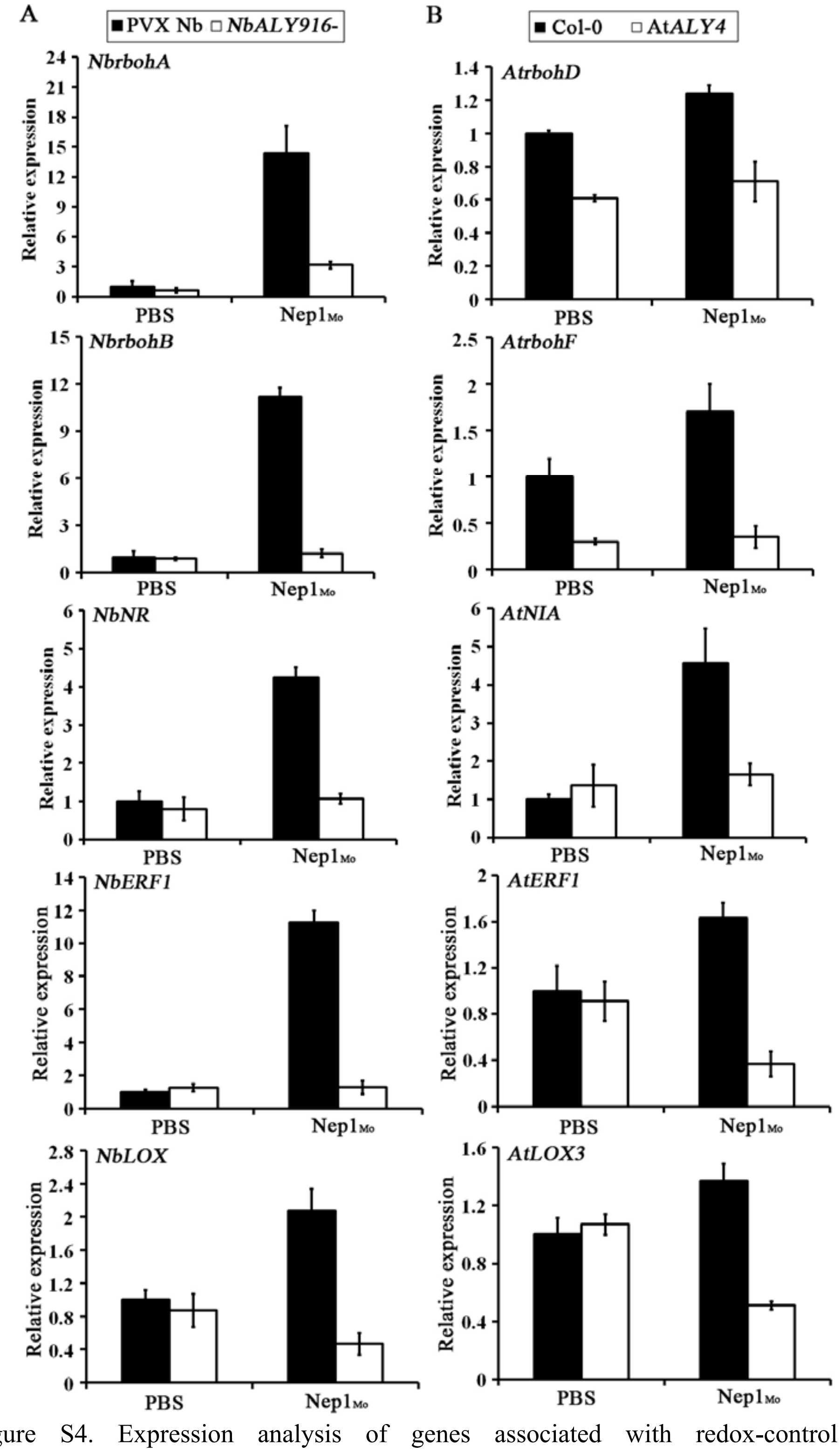

Figure S4. Expression analysis of genes associated with redox-control in *NbALY916*-silenced plants and At*ALY4* mutant by qRT-PCR. 6 h after treatment with or without $Nep1_{Mo}$ (50 nM), leaf samples were harvested from the inoculation site, the lower and the upper leaves; Bars represent mean (three technical replicates)±standard deviation.
